# End-of-Life Care Related Distress in the PICU and NICU: A Cross-Sectional Survey in a German Tertiary Center

**DOI:** 10.3389/fped.2021.709649

**Published:** 2021-09-24

**Authors:** Lars Garten, Andrea Danke, Tobias Reindl, Anja Prass, Christoph Bührer

**Affiliations:** ^1^Department of Neonatology, Charité–Universitätsmedizin Berlin, Berlin, Germany; ^2^Department of Vascular Surgery, Deutsches Rotes Kreuz Kliniken Berlin Köpenick, Berlin, Germany; ^3^Department of Pediatric Oncology and Hematology, Charité Universitätsmedizin Berlin, Berlin, Germany; ^4^Private Practice Dipl. Med. Trebuth, Beelitz, Germany; ^5^Department of Pediatric Pulmonology, Immunology and Intensive Care, Charité Universitätsmedizin Berlin, Berlin, Germany

**Keywords:** palliative care, newborn, children, intensive care unit, nurse, stress

## Abstract

**Objective:** To investigate and compare nurses' perceived care-related distress and experiences in end-of-life situations in neonatal and pediatric intensive care units.

**Study design:** Single-center, cross-sectional survey. Administration of an anonymous self-report questionnaire survey to nurses of two tertiary neonatal intensive care units (NICUs), and two tertiary pediatric intensive care units (PICUs) in Berlin, Germany.

**Results:** Seventy-three (73/227, response rate 32.2%) nurses completed surveys. Both, NICU (32/49; 65.3%) and PICU (24/24; 100.0%) nurses, reported “staffing shortages” to be the *most frequent* source of distress in end-of-life situations. However, when asked for the *most distressing* factor, the most common response by NICU nurses (17/49) was “lack of clearly defined and agreed upon therapeutic goals”, while for PICU nurses (12/24) it was “insufficient time and staffing”. No significant differences were found in reported distress-related symptoms in NICU and PICU nurses. The interventions rated by NICU nurses as most helpful for coping were: “discussion time before the patient's death” (89.6%), “team support” (87.5%), and “discussion time after the patient's death” (87.5%). PICU nurses identified “compassion” (98.8%), “team support”, “personal/private life (family, friends, hobbies)”, and “discussion time after the patient's death” (all 87.5%) as most helpful.

**Conclusions:** Distress-related symptoms as a result of end-of-life care were commonly reported by NICU and PICU nurses. The most frequent and distressing factors in end-of-life situations might be reduced by improving institutional/organizational factors. Addressing the consequences of *redirection of care*, however, seems to be a more relevant issue for the relief of distress associated with end-of-life situations in NICU, as compared to PICU nurses.

## Introduction

Most childhood deaths within the hospital, whether anticipated or unexpected, occur in settings primarily designed to provide acute, high-tech medicine intended for aggressive, often invasive, life-extending care: the neonatal intensive care unit (NICU) or the pediatric intensive care unit (PICU). Although palliative care services for children are becoming an accepted element of comprehensive, family-centered care (1, 2), the current nature of NICU and PICU setting presents special challenges to medical staff providing end-of-life care. Integrating psychosocial, spiritual, emotional, and practical support for families in addition to holistic treatment for the child might thus be perceived as representing merely another of many challenges.

Studies have shown that quality of patient care is reduced by increasing distress in nurses (3, 4). In caring for highly vulnerable intensive care unit (ICU) patients in end-of-life situations it is essential to evaluate specific factors of distress in nursing staff. Such evaluations have the potential to provide data necessary for (i) protecting ICU-nursing staff from emotional, psychological and physical harm by optimizing institutional support, and (ii) optimizing the quality of care of the dying ICU-patient.

Studies evaluating distress in medical staff caring for patients in end-of-life situations have been almost exclusively focused on adult (5–10), pediatric (11–18) *or* neonatal patients (19–28). There are only a very limited number of cross-sectional surveys (29–32) and almost all of them are directed specifically to “moral distress.”

Today, there exists a paucity of reliable information regarding nursing staff experiences with end-of-life care in German neonatal and pediatric intensive care units. As medical health care systems are different, results and conclusions from studies of other countries cannot be automatically applied to the local setting. The aim of the present work is to investigate and compare nurses' perceived care-related distress and experiences in end-of-life situations in tertiary neonatal and pediatric intensive care units of a German university hospital.

## Methods

### Setting

The survey was conducted at the Charité University Medical Center Berlin, Germany and comprised two tertiary neonatal intensive care units (NICUs), and two pediatric intensive care units (PICUs), a medical-surgical and a hematological/oncological, respectively. Eligible participants included all nurses involved in direct clinical care with at least 6 months of critical care experience.

### Questionnaire

A self-administered standardized questionnaire was used in order to (1) allow efficient collection of information from a large number of respondents; (2) eliminate interviewer-related errors and acquiescence bias; (3) allow the collection of a wide range of information; (4) allow participants to answer sensitive questions in private, and (5) permit ease of administration.

The questionnaire was written in German and contained four types of questions: (i) yes/no questions; (ii) multiple choice questions which allowed participants to select one or more answers; (iii) scaled questions featuring items to be rated using a five-point Likert scale with options such as “always” and “never” as the two extremes; and (iv) open ended questions.

The 18 questions used for this survey were adapted from previous reports (17, 33–35) or were newly written for the purposes of the present study. The face validity of the questionnaire was assessed by a four-person expert panel which consisted of one NICU nurse, one physician working in pediatric oncology, one member of a parents' psychological counseling team, and one psychologist with expertise in survey methodology. The panel reviewed all items for accuracy of content, comprehensiveness, and relevance to the aim of the present study. All panel members agreed that the content of the questionnaire was consistent with the objectives of the present work.

A preliminary version of the questionnaire was piloted with 26 participants of a multiprofessional panel composed of nurses, physicians and psychologists during a 4 week pediatric palliative care course at the Department of Children's Pain Therapy and Pediatric Palliative Care (Witten/Herdecke University, Faculty of Health–School of Medicine, Datteln, Germany), and questions were further revised based on the feedback.

The questionnaire acquired information on the following three topics:

1. Frequency and sources of distress perceived in end-of-life care situations.
2. Personal responses and coping mechanisms related to distress in end-of-life care situations.
3. Factors enabling staff to function effectively and cope with distress in end-of-life care.

All participants were also asked to provide basic sociodemographic information including age, gender, parental status, religion, years in intensive care, primary professional field (NICU or PICU), number of patients cared for in end-of-life situations, as well as experience with specialized training in palliative care, bereavement support and pain management/symptom control.

The data were collected in a manner that made it impossible to link a respondent to his or her response or to distinguish respondents from non-respondents.

### Data Collection

Nurses of the participating units were informed about the study via short lectures one month before being contacted by mail and asked to anonymously complete a posted paper version of the questionnaire. Eight weeks after posting the questionnaires a second appeal to participate in the study was sent by mail to all nurses. Inclusion of subsequently received questionnaires was limited to four additional weeks.

### Approval by Ethics Committee

The study was approved by the local institutional review board (Ethikkommission der Charité, #EA2/060/14).

### Statistical Analysis

All data were categorical in nature. The descriptive statistic used was the frequency distribution of each variable. The chi-square test was used to analyse proportions. All statistical calculations were made with the aid of SPSS version 19.0 (SPSS, Inc., Chicago, IL). The data from the three open-ended questions used in the survey were subjectively analyzed.

## Results

### Demographic Data

A total of 75/227 completed surveys were returned (2 were excluded for incomplete data), for a response rate of 32.2%. Of the remaining 73 surveys, 49 were from the NICU (response rate 36.6%, 49/134) and 24 were from the PICU (response rate 25.8%, 24/93). Respondents were predominately female (72 of 73, 98.6%), most were older than 40 years (46 of 73, 63.0%), and had more than 10 years of experience in critical care (50 of 73, 68.5 %). Sociodemographic data of all participating nurses are summarized in Table 1.

**Table 1:** Descriptive statistics of intensive care nurse participants.

	**NICU nurses**	**PICU nurses**
*n*	49	24
Age, y		
<30	6	4
30–40	9	8
>40	34	12
Sex (female), *n*	49	23
Have children, *n*	32	12
Religion, *n*		
Yes: No	17:32	8:16
ICU experience (years)		
<2	2	3
2–5	1	3
6–10	5	4
>10	36	14
No response	5	-
Participated in further training in		
Family-centered care on ICU	12	0
Palliative care	8	8
Bereavement care	34	8
Average number of patients per year cared for in end-of-life situations, *n*		
0	1	1
1–5	35	20
>5	2	3
No response	11	–
Time elapsed since last caring for a dying child (months)		
<6	16	17
6–12	16	4
>12	6	2
No response	11	1

### Frequency and Sources of Distress

#### Self-Reported Most Frequent Sources of Distress in End-of-Life Care Situations

**Figure 1:**
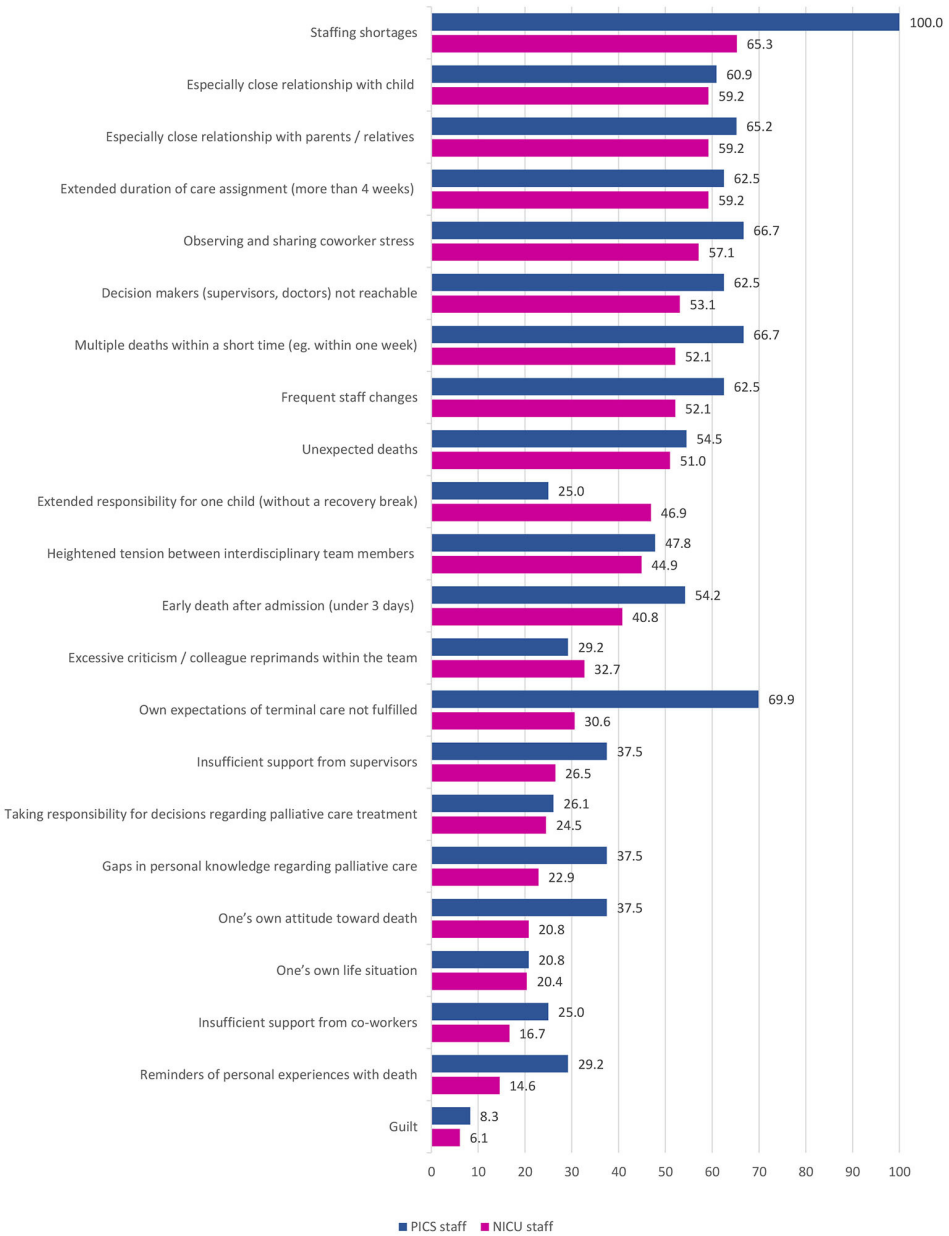
Self-reported **most frequent** (rated as “often,” “very often,” or “always”) sources of distress in end-of-life care situations as self-reported by NICU und PICU staff (%).

A list of 22 potential sources of distress in end-of-life care situations was presented and participants were asked to rate the frequency of each item using a five-point Likert scale with options “always” and “never” as the two extremes (*scaled question)*. Both, NICU (65.3%) and PICU (100.0%) nurses, reported “staffing shortages” to be the most frequent source of distress in end-of-life situations. Additional distressing factors frequently reported by NICU nurses included “especially close relationship with the child”, “especially close relationship with parents/relatives”, and “extended duration of care assignment (more than 4 weeks)”, all at identical rates of 59.2%. PICU nurses also rated “own expectations of terminal care not fulfilled” (69.6%), “multiple deaths within a short time (e.g. within one week)”, and “observing coworkers being in distress”, the latter two in 66.7% of respondents, as very frequent sources of distress. Self-reported most frequent sources of distress in end-of-life care situations by NICU und PICU staff are summarized in Figure 1.

#### Self-Reported Most Distressing Factors in End-of-Life Care Situations

When openly asked for the most distressing factors in end-of-life situations with a maximum of 5 answers allowed, the answers of NICU and PICU nurses divided into three main groups: distress related to (i) the dying child, (ii) institutional/organizational factors, and (iii) the parents.

The three most frequently reported distressing factors were “lack of clearly defined and agreed upon therapeutic goals” (17/49), “pain” (14/49), and “dyspnea” (12/49) for NICU nurses, and “insufficient time and staffing” (12/24), “pain” (10/24), and “dyspnea” (10/24) for PICU nurses, respectively. Self-reported most distressing sources of distress in end-of-life care situations by NICU und PICU staff are summarize in Table 2.

**Table 2:** Self-reported most distressing sources of distress in end-of-life care situations by NICU and PICU staff.

	**NICU nurses, *n* = 49**	**PICU nurses, *n* = 24**
	***N***	***N***
**Factors related to the child**		
Pain	14	10
Dyspnea	12	10
Children's agitated emotional distress	4	8
Seizures	4	2
Edema	3	–
Sleep disturbances	–	2
Extended course of illness	6	4
**Institutional/organizational/personal factors [systemic (policy and procedure) factors]**		
Insufficient time and staffing	10	12
Lack of clearly defined and agreed upon therapeutic goals	17	1
Unclear communication	4	–
One's own thoughts and feelings	7	3
Inadequate support	4	–
Inexperience	3	2
Conflicts and disagreements	2	1
Nursing unit disorganization	5	2
Dealing with the topic of death on the unit	–	3
Inadequate workspace	–	2
Daily unit stress	1	1
**Factors related to of the parents**		
Explanations and conversations	7	3
Parental behavior	6	3
Parental psychological state	5	4
Parents' personal situation	3	1
Language barriers	1	4

*Open questions, up to five answers were allowed per participant*.

### Self-Reported Distress-Related Symptoms and Coping Mechanisms

#### Self-Reported Distress-Related Symptoms as a Result of End-of-Life Care

A list of 22 distress-related reactions or symptoms was presented and participants were asked to rate the frequency of each item using a five-point Likert scale with options “always” and “never” as the two extremes. The most frequent (rated as “often,” “very often,” or “always”) distress-related reactions or symptoms reported by NICU nurses were: (i) “Excessive self-criticism” (33.3%), (ii) “Emotional exhaustion or symptoms” (33.3%), and (iii) “Speechlessness” (27.2%). PICU nurses identified “Emotional exhaustion or symptoms” (58.4%), “Sleep difficulty/upsetting dreams” (50.0%), and “Irritability” (both 45.9%) as most frequent distress-related reactions or symptoms. No significant differences in frequency of self-reported distress-related symptoms as a result of end-of-life care were found.

**Figure 2:**
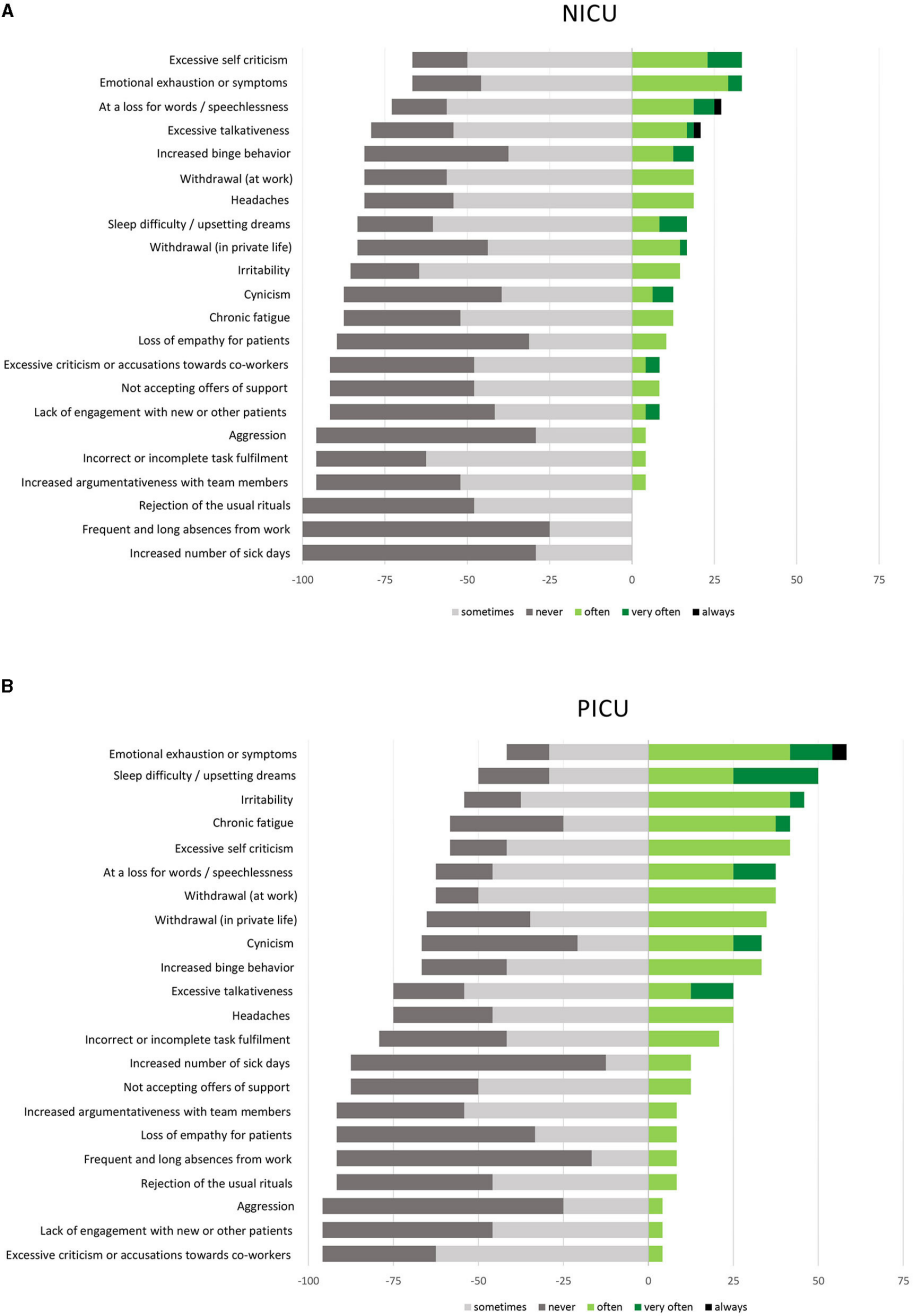
Most frequent distress-related reactions or symptoms as self reported by **(A)** NICU staff and by **(B)** PICU staff (%). All questions to be rated by using a five-point Likert Scale.

Self-reported most frequent distress-related reactions or symptoms as a result of end-of-life care by NICU und PICU staff are summarize in Figures 2A,B.

#### Coping Mechanisms in End-of-Life Care

A list of 15 helpful mechanisms for coping in or after end-of-life care situations was presented and participants were asked to rate the efficacy of each item using a five-point Likert scale with options “helps very well” and “never helps” as the two extremes. In addition, also “not available/no experience” was an optional answer.

**Figure 3:**
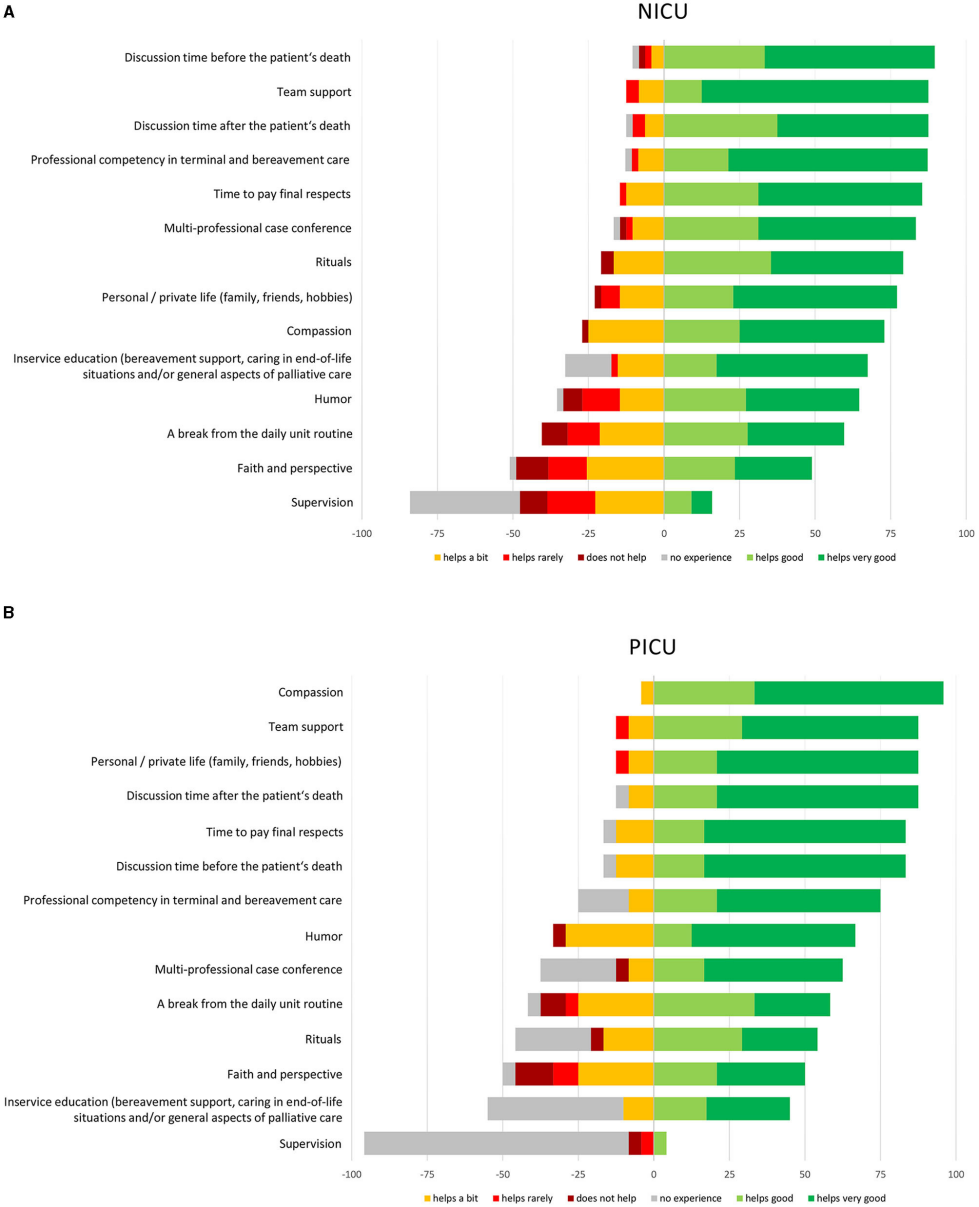
Coping mechanisms in end-of-life care as self reported by **(A)** NICU staff and by **(B)** PICU staff (%). All questions to be rated by using a six-point Likert Scale.

The mechanisms rated as most helpful (rated as “helps good” or “helps very good”) for coping in or after end-of-life care situations reported by NICU nurses were: “discussion time before the patient's death” (89.6%), “team support” (87.5%), and “discussion time after the patient's death” (87.5%). PICU nurses identified “compassion” (98.8%), “team support”, “personal/private life (family, friends, hobbies)”, and “discussion time after the patient's death” (all 87.5%) as the most helpful coping mechanisms. Self-reported most helpful coping mechanisms in or after end-of-life care situations by NICU und PICU staff are shown in detail in Figures 3A,B.

## Discussion

To our knowledge, this is the first cross-sectional survey in tertiary neonatal and pediatric intensive care units in Germany investigating and comparing nurses' perceived care-related distress and experiences in end-of-life situations.

There was no difference in the distress factors to which the nurses felt most frequently exposed. Both neonatal and pediatric nurses identified “staff shortages” as the most prominent factor. The nurses at the PICUs additionally identified this as the source of their greatest personal distress. The NICU nurses, however, ranked “lack of staff and time” as fourth among the “self-reported most distressing factors in end-of-life care situations”.

The NICU nurses, however, felt the greatest personal distress when the precise treatment goal for the patients they cared for had not been clearly defined, for example, due to the unclear prognosis.

In contrast, this factor was only named by one PICU nurse. The issue of changing therapeutic goals during the process of inpatient intensive care thus seems to be NICU-specific. In a previous study (36), we found that 4/5 neonatal palliative patients investigated (*n* = 149) were initially treated with a curative therapeutic goal. Only in the course of treatment did the therapeutic goal change from curative to palliative. In comparison, the proportion of pediatric patients converting from an initially curative treatment approach to a subsequent palliative therapeutic goal as a proportion of the total pediatric palliative population lies at ~25–33% (37, 38).

With regard to symptoms in childhood end-of-life care situations no differences could be found in the personal distress assessment by the nurses. Inadequately treated pain and dyspnea were named as most distressing factors in end-of-life care situations by both NICU and PICU nurses.

Distress-related personal symptoms as a result of end-of-life care were commonly reported by German NICU and PICU nurses. The overall frequency of self-reported symptoms was not significantly different in both groups. Coping with distress in end-of-life situations was best facilitated by having enough time for communication with parents and within the team, and by receiving collegial support and empathy from coworkers or family/friends. When available, in-service education was rated as helpful or very helpful by nearly all NICU and PICU nurses. In nursing education, information on psychological distress related to children's deaths and bereavement care should be conveyed from the early stage.

There are two main limitations to this questionnaire study. First, reporting bias is difficult to totally prevent or estimate. Second, we present data from a single center and cannot be sure that our findings are applicable to the entire population outside of our institution.

In conclusion, distress-related personal symptoms as a result of end-of-life care were commonly reported by German NICU and PICU nurses. The most frequent and distressing factors in end-of-life situations might be reduced by improving institutional/organizational factors (e.g., appropriate levels of staffing and provision of sufficient time for communication). However, addressing the specific professional, moral and emotional consequences of *redirection of care* seems to be a more relevant issue for the relief of distress associated end-of-life situations in the NICU compared to the PICU.

## Data Availability Statement

Anonymized data will be provided to researchers with a project approved by an institutional review board after consultation with the data protection officer.

## Ethics Statement

The studies involving human participants were reviewed and approved by the local institutional review board (Ethikkommission der Charité Universitätsmedizin Berlin, #EA2/060/14). By voluntarily sending the anonymous self-report questionnaire survey back to the research group the participants provided consent to participate in this study.

## Author Contributions

LG, AD, and CB conceptualized the study and analyzed the data. LG, AD, TR, and AP collected the data. LG wrote the first draft of the manuscript. All authors contributed to the interpretation of the data and critically reviewed and contributed to the final draft of the manuscript.

## Funding

We acknowledge support from the Open Access Publication Fund of Charité–Universitätsmedizin Berlin. LG is participant in the BIH-Charité Clinical Fellows Program funded by the Charité–Universitätsmedizin Berlin and the Berlin Institute of Health (BIH).

## Conflict of Interest

The authors declare that the research was conducted in the absence of any commercial or financial relationships that could be construed as a potential conflict of interest.

## Publisher's Note

All claims expressed in this article are solely those of the authors and do not necessarily represent those of their affiliated organizations, or those of the publisher, the editors and the reviewers. Any product that may be evaluated in this article, or claim that may be made by its manufacturer, is not guaranteed or endorsed by the publisher.

## Abbreviations

NICU: neonatal intensive care unit
PICU: pediatric intensive care
ICU: intensive care.
